# Supplementary figures and images for: Concurrent decoding of distinct neurophysiological fingerprints of tremor and bradykinesia in Parkinson’s disease
Source: eLife. 2023 May 30;12:e84135. doi: 10.7554/eLife.84135 (PMC10264071; doi:10.7554/eLife.84135)

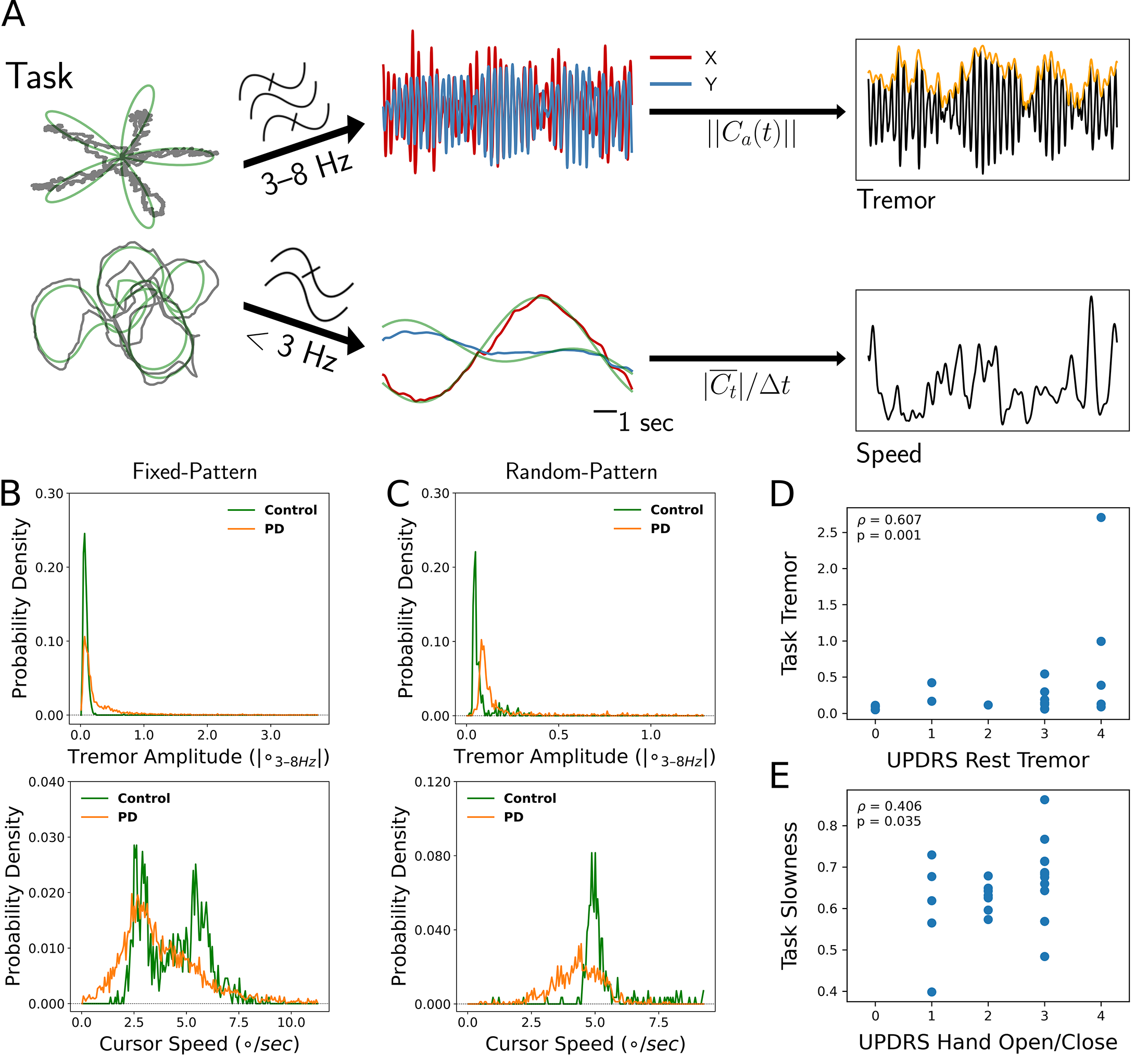

Supplement: MDAR checklist [file elife-84135-mdarchecklist1.zip › fig1.tif]

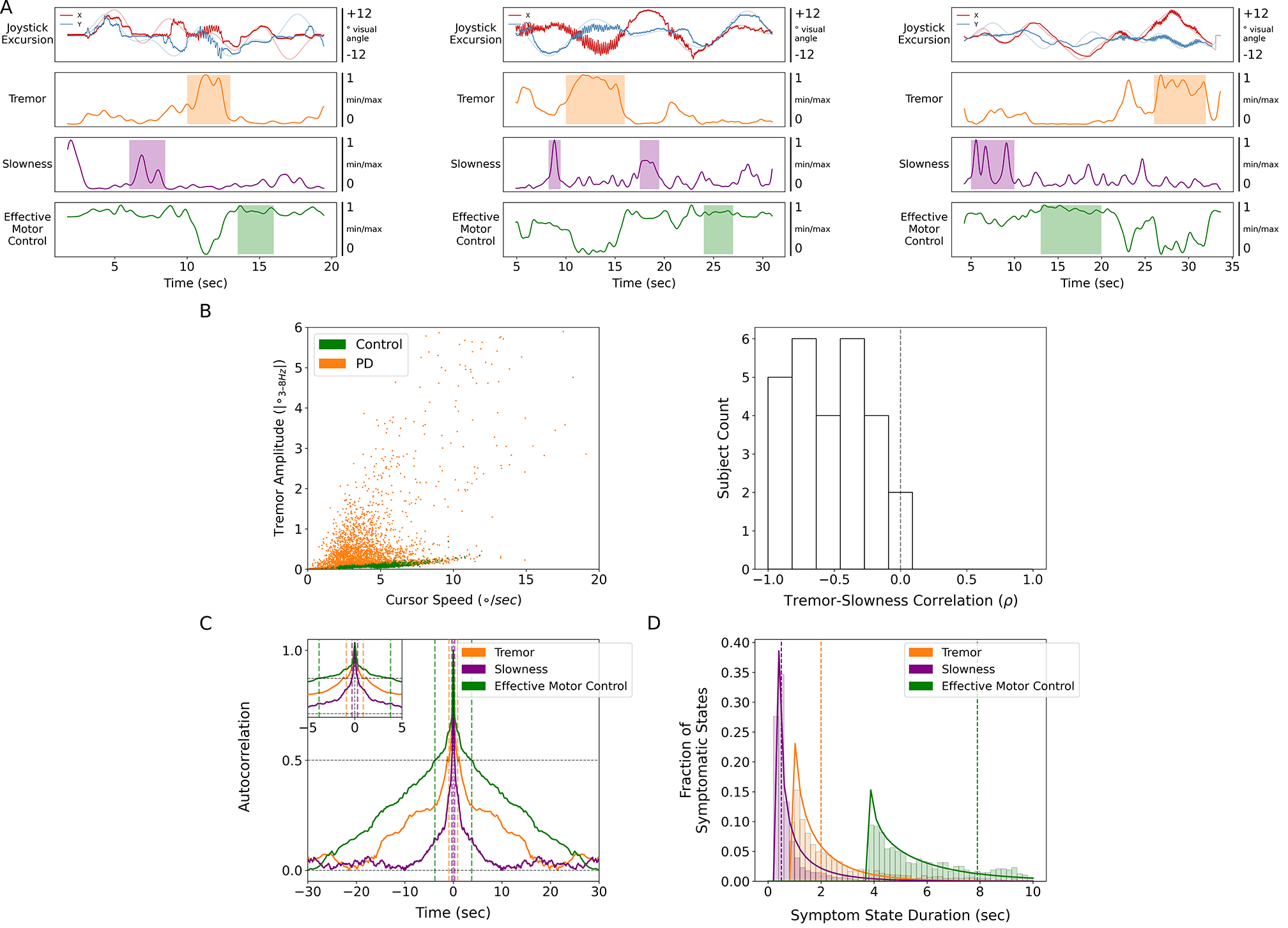

Supplement: MDAR checklist [file elife-84135-mdarchecklist1.zip › fig2.tif]

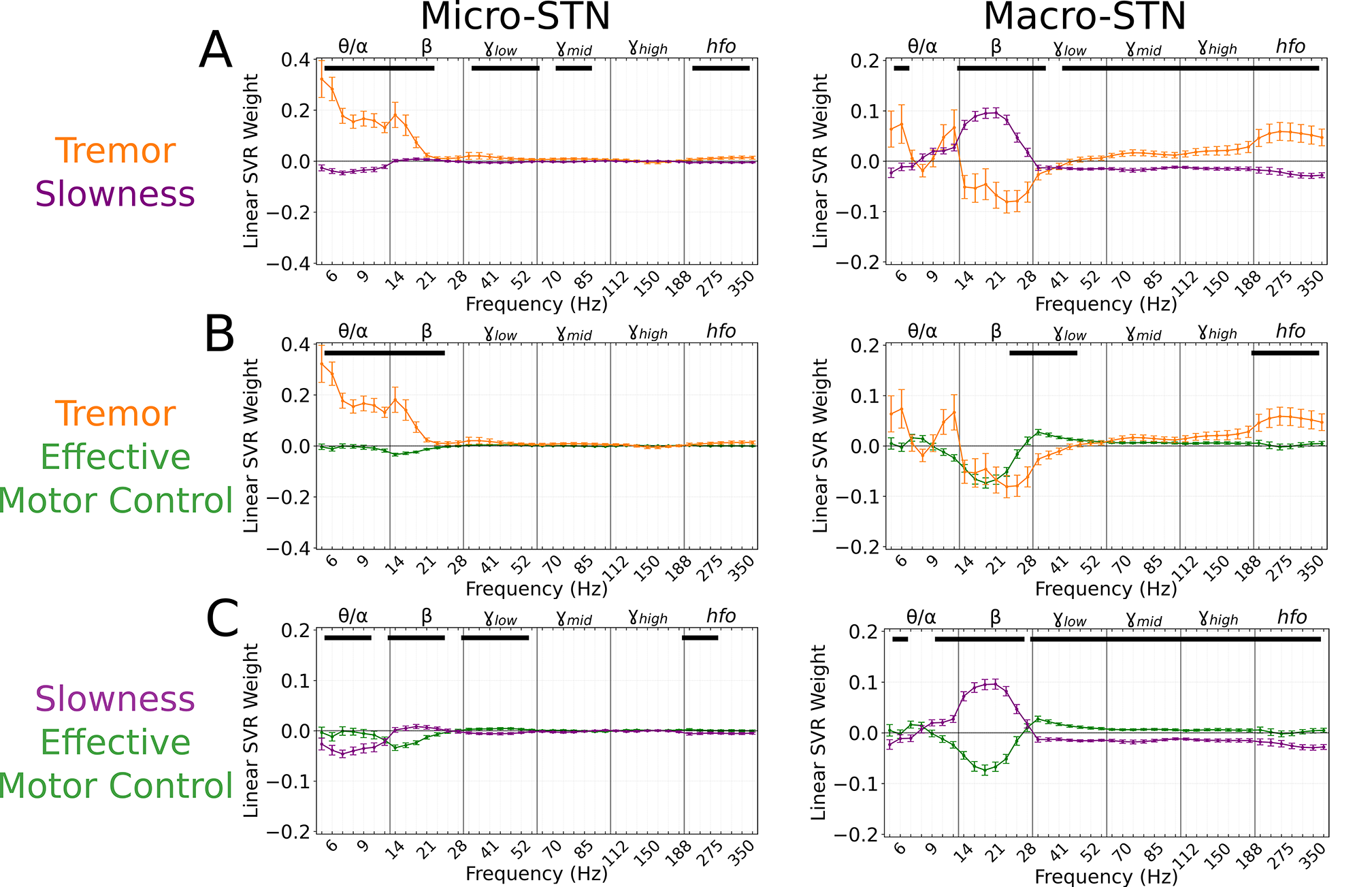

Supplement: MDAR checklist [file elife-84135-mdarchecklist1.zip › fig3.tif]

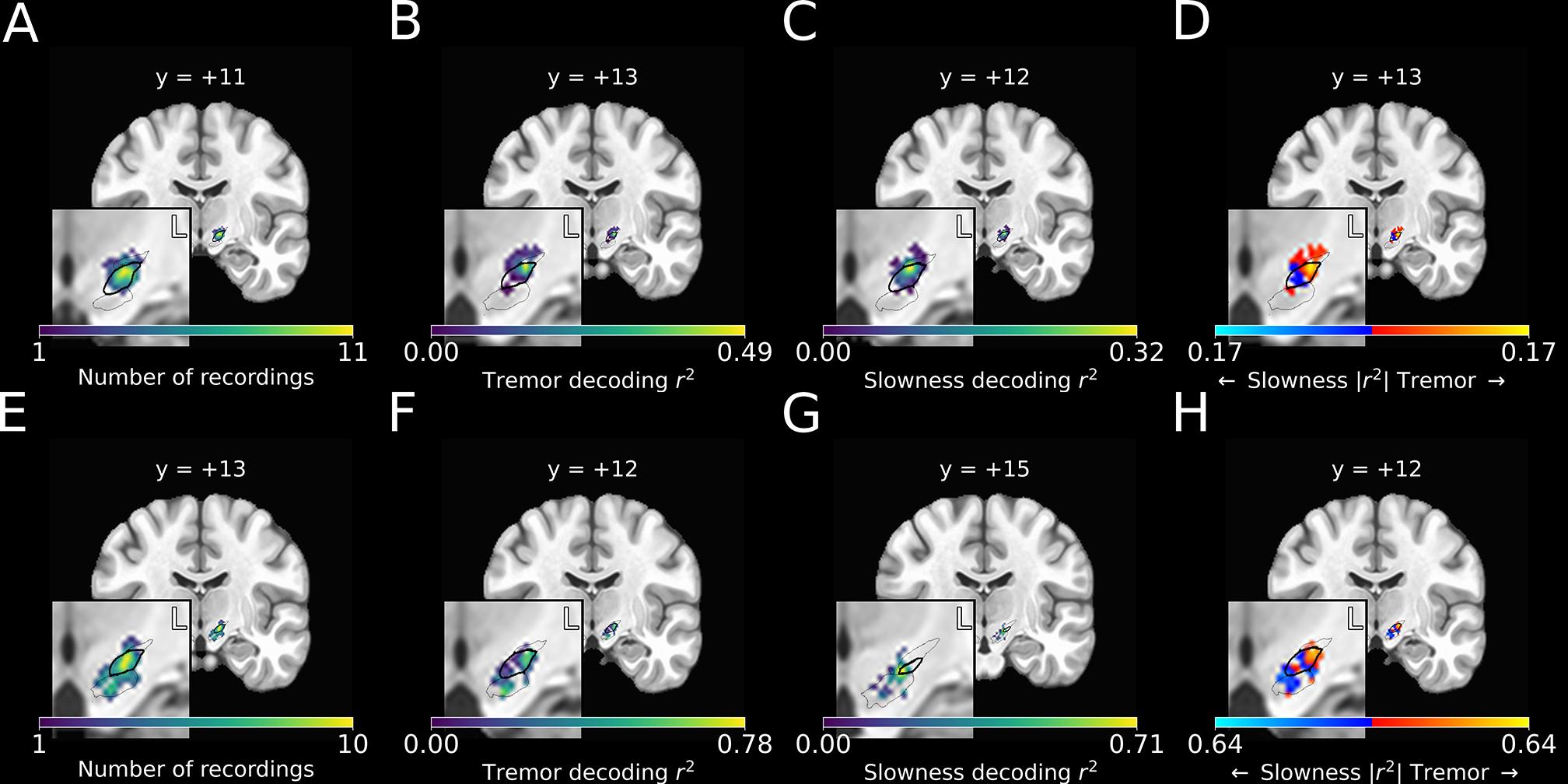

Supplement: MDAR checklist [file elife-84135-mdarchecklist1.zip › fig4.tif]

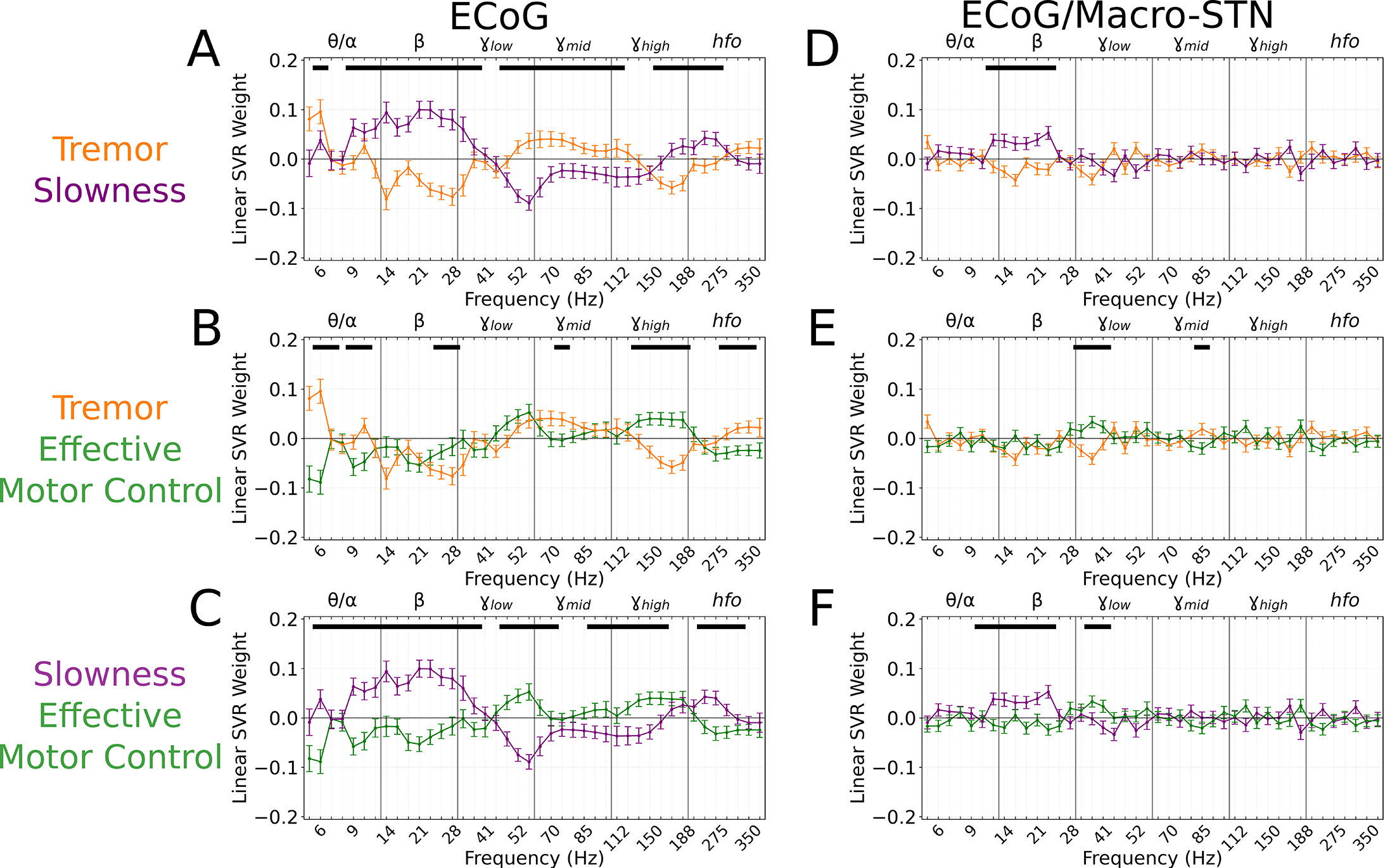

Supplement: MDAR checklist [file elife-84135-mdarchecklist1.zip › fig5.tif]

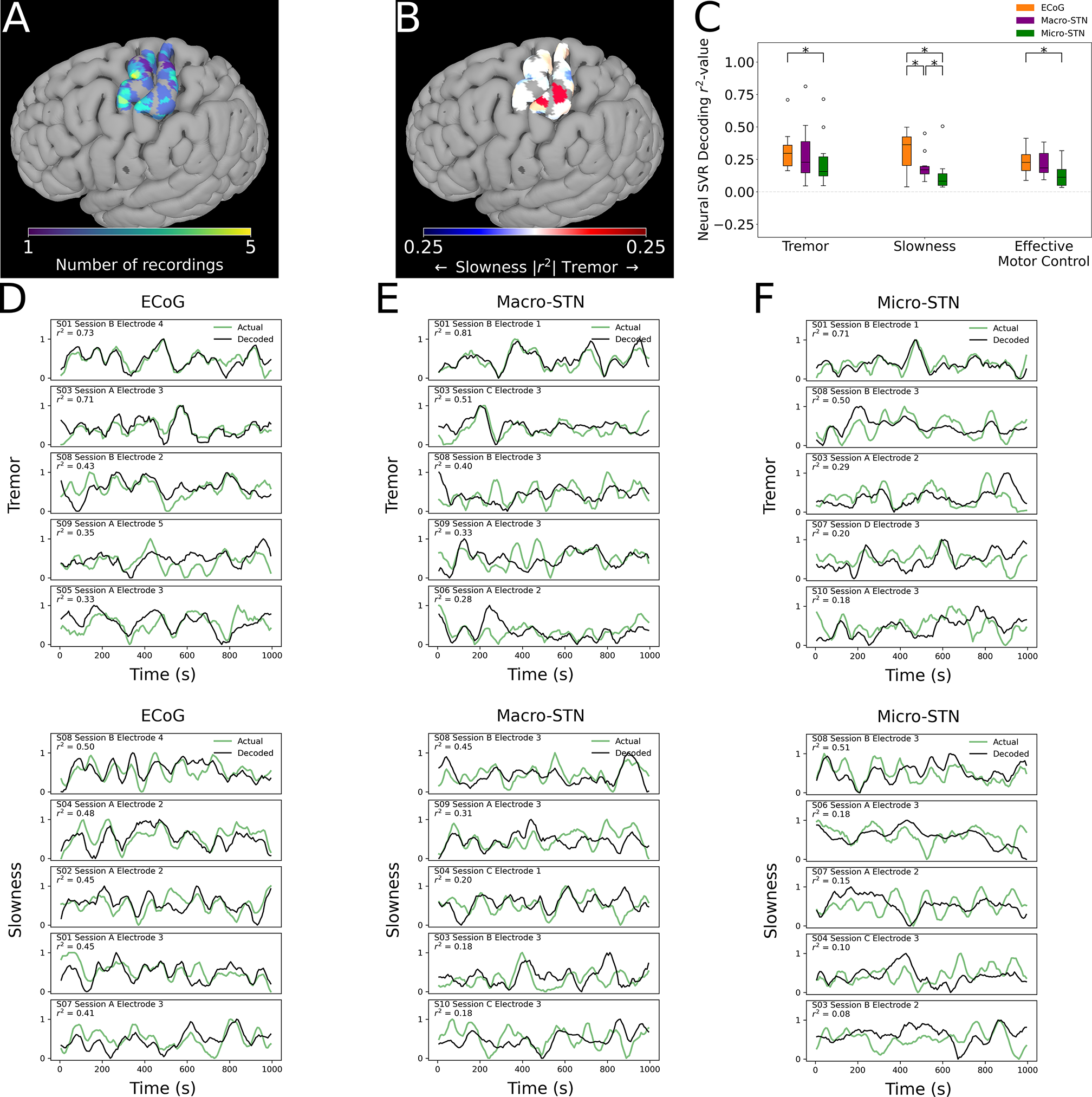

Supplement: MDAR checklist [file elife-84135-mdarchecklist1.zip › fig6.tif]
